# Boundary conditions of collaborative learning using worked-examples: joint moderating effects of task-specific prior knowledge and task complexity on transfer performance and cognitive load

**DOI:** 10.3389/fpsyg.2026.1855051

**Published:** 2026-06-23

**Authors:** Ying Wang, Qiong Li, Xiping Liu

**Affiliations:** 1 Faculty of Psychology, Tianjin Normal University, Tianjin, China; 2 Qiqihar Medical University, Qiqihar, China; 3 Jiangxi Environmental Engineering Vocational College, Ganzhou, China; 4 School of Sociology, University of Sanya, Sanya, China

**Keywords:** cognitive load, collaborative learning, prior knowledge, task complexity, worked-examples

## Abstract

When collaborative learning facilitates learning, remains a central question in educational research. Grounded in cognitive load theory, this study examined whether the effectiveness of collaborative, relative to individual, learning from worked-examples is jointly moderated by learners’ experimentally induced task-specific prior knowledge and task complexity. A 2 (task-specific prior knowledge: low vs. high) × 2 (task complexity: low vs. high) × 2 (learning condition: individual vs. collaborative) mixed design was employed, with task complexity as a within-subjects factor. A total of 134 fifth-grade students participated in the study. They studied mathematical worked-examples of varying complexity, either individually or collaboratively, and then completed near-transfer and far-transfer tests as well as cognitive load ratings. Results showed a significant three-way interaction among task-specific prior knowledge, task complexity, and learning condition for near-transfer, indicating that the effect of learning condition depended jointly on learners’ task-specific prior knowledge and task complexity. Specifically, under high task complexity, collaborative learning outperformed individual learning among learners with high task-specific prior knowledge, whereas no stable collaborative advantage was found for learners with low task-specific prior knowledge. For far-transfer, the main effects of task-specific prior knowledge and task complexity were significant, whereas the main effect of learning condition and all interaction effects did not reach statistical significance. Thus, collaborative learning did not show a stable advantage in far-transfer tasks. The cognitive load results provided supportive evidence for understanding the differences in transfer performance: students in the collaborative learning condition reported lower subjective cognitive load than those in the individual learning condition, and high complexity tasks elicited higher cognitive load than low complexity tasks. Overall, the findings suggest that the effectiveness of collaboration in learning from worked-examples is conditional rather than universal. Its benefits depend on the fit between task complexity and learners’ task-specific prior knowledge. The study provides empirical evidence on the boundary conditions of collaborative learning in elementary mathematics instruction through worked examples and contributes to a better understanding of when collaborative learning is most effective.

## 1 Introduction

Collaborative learning has become a widely used instructional strategy (Zambrano et al., 2019), often serving as an umbrella term for a broad range of group-based instructional approaches (Yang, 2023). Typically, it refers to learning contexts where students work together in small groups to solve problems and develop skills (Zambrano et al., 2023). There is a common assumption among educators and the general public that collaborative learning is inherently superior to individual learning (Nokes-Malach et al., 2015). This belief has strongly shaped instructional practices across educational levels from K–12 to higher education and continues to influence teaching in diverse cultural contexts worldwide (Kollias et al., 2005). However, empirical evidence does not consistently support the superiority of collaboration for learning outcomes. In fact, research has shown that, in some cases, collaborative learning may be less effective than individual learning or may not differ significantly from it (Ashman et al., 2020; Retnowati et al., 2018; Loibl et al., 2017; Slavin, 2014; Kirschner et al., 2011; Kirschner et al., 2009a). Overall, these findings indicate that the effectiveness of collaborative learning is not universal but depends critically on the conditions under which it is implemented (Kirschner et al., 2018).

Cognitive load theory (CLT) provides a useful theoretical framework for understanding this issue. As an instructional theory grounded in human cognitive architecture, CLT assumes that learning is constrained by the limited capacity of working memory (Cowan, 2014; Sweller et al., 2019; Sweller, 2021, 2024). Because working memory can process only a limited amount of information at any one time, unnecessary additional demands imposed on the cognitive system increase cognitive load and may hinder both learning and transfer (Sweller et al., 2019). From this perspective, collaborative learning fundamentally changes how cognitive load is distributed during learning. On the one hand, collaboration may enhance learners’ capacity to deal with complex tasks through the collective working memory effect (Kirschner et al., 2009a). On the other hand, it may also impose additional extraneous load due to the communication and coordination demands involved in group interaction, thereby undermining learning (Kirschner et al., 2009a, 2009b). The effectiveness of collaborative learning, therefore, depends on whether its cognitive benefits outweigh its coordination costs. Importantly, this balance is likely to be influenced by two key variables, namely learners’ prior knowledge and task complexity. More generally, the effects of collaborative learning are closely related to learner characteristics and the nature and complexity of the task. When these factors and their interplay are not taken into account, the effectiveness of collaborative learning becomes difficult to predict and is likely to appear unstable (Kirschner et al., 2018).

Within cognitive load theory, prior knowledge is regarded as a key variable influencing learning outcomes, and its role is often discussed in relation to the expertise reversal effect (Kalyuga, 2007; Jiang et al., 2023). Prior research has shown that novice learners typically require more external support and structured guidance, whereas more knowledgeable learners may find such support redundant (Kalyuga, 2007; Janssen and Kirschner, 2020). This difference is rooted in the knowledge structures available in long-term memory (Sweller, 2024). Learners with greater expertise can draw on a richer knowledge base to process complex tasks more efficiently despite the limitations of working memory, whereas novices are more reliant on their limited working memory during problem solving. Accordingly, whether collaborative learning improves learning outcomes may depend to a considerable extent on learners’ prior knowledge (Mende et al., 2020; Gui et al., 2025).

Within the framework of cognitive load theory, task complexity is a core factor influencing performance and behavior in collaborative learning (Chen et al., 2023). Task complexity is an inherent characteristic of the task itself and reflects the amount of cognitive resources required to complete a learning task as well as the degree of interdependence among task elements (Sweller et al., 2019; Endres et al., 2022; Sweller, 2023). When a task contains many interacting elements, learners must process and integrate multiple units of information simultaneously in working memory, thereby imposing high intrinsic cognitive load. Previous studies have suggested that collaborative learning is more likely to be advantageous for high complexity tasks (Chen et al., 2018; Järvelä et al., 2020). According to the collective working memory effect, group members can distribute cognitive processing across individuals through division of labor and shared processing, thereby overcoming the working memory limitations of a single learner (Sweller et al., 2019). In such cases, the benefits of collective working memory may exceed transaction costs, thereby facilitating effective learning (Jiang and Kalyuga, 2022). By contrast, in low complexity tasks, collaboration often introduces additional coordination load, namely the cognitive resources consumed during communication, negotiation, and synchronization among group members (Kirschner et al., 2009b). These costs may offset the potential benefits of collaboration. Taken together, task complexity may either elicit the group advantage of collaborative learning or undermine its effectiveness because of increased coordination costs. It is therefore necessary to further examine how the benefits of collaborative learning vary across levels of task complexity and how such variation affects transfer.

Worked-examples are widely regarded as an effective instructional approach for promoting knowledge construction because they directly present both the procedural steps and the underlying principles of problem-solving (Renkl, 2014; Chen et al., 2021). However, when worked-examples are used in collaborative settings, a puzzling contradiction emerges. On the one hand, theoretical accounts suggest that collaboration should help reduce the burden imposed by cognitively demanding tasks through the collective working memory effect (Kirschner et al., 2018). On the other hand, key empirical studies have reported the opposite pattern. Retnowati et al. (2018) found that students who studied worked- examples individually achieved significantly better learning outcomes than those who studied them collaboratively. Interpreting this finding from the perspective of evolutionary educational psychology, the authors argued that collaboration, as a biologically primary skill (Geary, 2008, 2012), may direct learners’ cognitive resources toward social coordination, thereby reducing the deep processing of biologically secondary knowledge that requires deliberate practice (Geary, 2008, 2012). Under such conditions, collaboration may become redundant rather than beneficial. This pattern suggests that the benefits of collaborative learning are subject to an important boundary condition.

Taken together, the literature reviewed above suggests that collaborative learning, as a complex learning activity, may be jointly influenced by multiple factors, and that its effectiveness is therefore highly condition-dependent (Kirschner et al., 2018). However, two main limitations remain in the existing literature. First, many prior studies have examined pairwise relations among variables relevant to collaborative learning. For example, Kirschner et al. (2011) examined how task complexity moderated the relative efficiency of individual and collaborative learning; Zambrano et al. (2019) investigated how prior knowledge influenced individual and collaborative learning outcomes; and Retnowati et al. (2018) compared individual and collaborative learning in the context of learning mathematics from worked-examples. Although these studies have provided important evidence on the conditional effectiveness of collaborative learning, relatively limited attention has been paid to the joint effects of prior knowledge, learning condition, and task complexity within an integrated experimental framework. Second, in basic education contexts, fine-grained research examining how these variables jointly influence near-transfer, far-transfer, and cognitive load remains scarce. Kaplan (2023) further argued that educational psychology research should move beyond traditional paradigms by paying closer attention to the role of educational policy and authentic educational contexts in shaping the phenomena under investigation. Incorporating policy and real educational contexts into research would not only enhance the field’s practical relevance but also constitute an important step toward developing theories with greater ecological validity.

In summary, the effectiveness of collaboration in learning from worked-examples is unlikely to be universal and may instead depend on how well learners’ prior knowledge aligns with task complexity. Drawing on cognitive load theory, the present study investigated whether, under worked-example conditions, the relative benefits of collaborative over individual learning increase as task complexity rises, and whether learners’ task-specific prior knowledge further moderates this effect. Three hypotheses were proposed, H1: For near-transfer, an interaction between task-specific prior knowledge, task complexity, and learning condition is expected, with high task-specific prior knowledge learners benefiting more from collaboration under high complexity tasks. H2: For far-transfer, collaborative learning is not universally advantageous but is expected to be more beneficial under high complexity tasks. H3: For cognitive load, greater task complexity is predicted to increase subjective cognitive load, especially for learners with low task-specific prior knowledge. By jointly examining transfer performance and subjective cognitive load, this study seeks to clarify the boundary conditions for effective collaborative learning from worked-examples and to provide nuanced empirical guidance on the appropriate use of collaborative activities in elementary mathematics instruction.

## 2 Methods

### 2.1 Experimental design

The study utilized a 2 (task-specific prior knowledge: low vs. high) × 2 (learning condition: individual vs. collaborative) × 2 (task complexity: low vs. high) mixed factorial design. Task-specific prior knowledge and learning condition served as between-subjects factors, while task complexity was a within-subjects factor. The dependent variables included near-transfer performance, far-transfer performance, and cognitive load.

### 2.2 Participants

An *a priori* power analysis conducted with G*Power (*f* = 0.25, *α* = 0.05, 1 − *β* = 0.80) indicated a minimum sample size of 128 participants. Four parallel fifth-grade classes from an urban public elementary school were recruited as the initial sample. The mean scores of these classes on the most recent standardized mathematics examination were 85.66, 86.27, 85.52, and 86.83, respectively. A one-way analysis of variance found no significant differences among the classes, suggesting that students’ prior mathematical knowledge was comparable across groups and the sample was appropriate for group assignment.

After screening, 134 students completed the experiment (47.6% boys, 52.4% girls), ranging in age from 10 to 12 years, consistent with typical fifth-grade students. Following the pretest, students who proceeded to the main experiment received different preparatory instruction to establish varying levels of task-specific prior knowledge. Of the 134 participants, 67 students were assigned to the high task-specific prior knowledge condition and 67 students were assigned to the low task-specific prior knowledge condition. Within the high task-specific prior knowledge condition, 32 students were assigned to individual learning and 35 to collaborative learning. Within the low task-specific prior knowledge condition, 35 students were assigned to individual learning and 32 to collaborative learning. Thus, the four experimental groups were approximately balanced in size. The study was reviewed and approved by the Ethics Committee, and written informed consent was obtained from both the students and their parents prior to participation.

### 2.3 Materials

The experimental materials included pretest materials, preparatory instructional materials, worked-examples, transfer test materials, and a cognitive load questionnaire. To ensure the content validity of the research instruments, the pretest materials, preparatory instructional materials, worked-examples, and transfer test items were all adapted or developed based on the compulsory elementary mathematics textbooks published by the People’s Education Press. The content focused on the area calculation of triangles, parallelograms, and trapezoids, which is consistent with the mathematics curriculum and learning experience of fifth-grade students.

*Pretest materials*: The pretest consisted of 10 items and was used to determine participants’ eligibility for the study. The first seven items assessed students’ previously acquired foundational knowledge of polygon perimeters and areas, whereas the final three items assessed area formulas for triangles, parallelograms, and trapezoids that had not yet been formally taught.

*Preparatory instructional materials*: The preparatory instructional materials were divided into a high task-specific prior knowledge set and a low task-specific prior knowledge set. The high task-specific prior knowledge materials included formula derivation for triangles, parallelograms, and trapezoids; one simple worked example for the area calculation of each of these three shapes; and one complex worked example for the area calculation of each of these three shapes. All worked examples contained the following components: “given,” “find,” “solution,” “analysis,” “formula,” and “calculation steps.” In the simple worked-examples, students were required to calculate polygon areas by directly applying the basic formulas. In the complex worked-examples, students were required to complete a one-step derivation based on transformed formulas before calculating the area. The low task-specific prior knowledge materials consisted of one instructional set on deriving the area formulas for triangles, quadrilaterals, and trapezoids.

*Worked-example materials*: The worked-example materials were divided into low and high complexity worked-examples. The low complexity worked-examples included one simple example for each of the following: triangle area calculation, parallelogram area calculation, and trapezoid area calculation. These examples required students to calculate polygon areas by directly applying the basic formulas. The high complexity worked-examples likewise included one example for each type of complex area calculation. In these examples, one condition was left unknown during the solution process, requiring students to first infer the missing information from the other given conditions and then apply the polygon area formula to complete the calculation. Both types of worked examples contained the following components: “given,” “find,” “solution,” “analysis,” “formula,” and “calculation steps”.

*Transfer test materials*: The transfer test materials corresponded to the low and high complexity worked-examples, respectively. Each set included a near-transfer test and a far-transfer test. The near-transfer items were structurally isomorphic to the worked-examples and assessed whether students could apply the same formulas and solution procedures to similar problems. The far-transfer items required students to apply the same underlying area-calculation principles to structurally different or more complex problem contexts. The scoring rubrics awarded points for correct formula application, key solution steps, and correct numerical answers, thereby assessing not only final performance but also the application of relevant mathematical procedures. Under the low complexity condition, the transfer test consisted of six polygon-area problems, including three near-transfer items and three far-transfer items. The near-transfer items were structurally isomorphic to the worked examples, whereas the far-transfer items required students to apply the derived formulas from the original worked examples to solve new area-calculation problems. Under this condition, internal consistency was Cronbach’s *α* = 0.679 for the near-transfer test and Cronbach’s α = 0.685 for the far-transfer test. Under the high complexity condition, the transfer test likewise consisted of six items: three near-transfer items and three far-transfer items. The near-transfer items were structurally isomorphic to the worked- examples, whereas the far-transfer items involved calculations of polygon areas for two composite figures. Under this condition, internal consistency was Cronbach’s α = 0.621 for the near-transfer test and Cronbach’s *α* = 0.758 for the far-transfer test.

The materials were reviewed by mathematics teachers and educational psychology researchers to examine whether the items were appropriate for fifth-grade students, adequately represented the intended learning content, and clearly distinguished near-transfer from far-transfer tasks as well as low complexity from high complexity tasks. Revisions were made to item wording, figure presentation, and scoring rubrics based on the feedback.

*Cognitive load measure*: cognitive load was measured using the 9-point Paas Cognitive Load Scale (Paas, 1992; van Gog and Paas, 2008), which includes two items: one assessing students’ invested mental effort during learning and another evaluating their subjective perception of the material’s difficulty. The items were: “How much mental effort did you invest in the learning process just now?” and “How difficult did you think the learning materials were just now?” Previous research has demonstrated that the scale possesses good reliability (Cronbach’s *α* ≈ 0.90; test–retest *r* ≈ 0.88). It should be noted that perceived difficulty was treated as a subjective indicator of cognitive load rather than as a direct measure of task complexity. Task complexity was manipulated a prior through the structural features of the worked-examples. The mean of the two items was used as the subjective cognitive load score.

### 2.4 Procedure

The experiment consisted of five stages: pretest, task-specific prior knowledge instruction, group assignment, learning from worked-examples of varying task complexity, and transfer testing.

*Pretest*: participants individually completed a paper-based pretest under standardized instructions, which lasted 10 min. Students who answered the first seven items correctly or partially correctly but could not solve the final three items were selected for the main experiment. The final three items assessed target knowledge that had not yet been formally taught and would be addressed in the subsequent preparatory instruction and worked-example learning phases. Therefore, students who solved these items were excluded because they had already acquired the target knowledge, which could reduce the validity of the prior knowledge manipulation.

*Preparatory instruction*: to induce different levels of task-specific prior knowledge, two intact classes were randomly assigned to the high task-specific prior knowledge instruction condition and the other two to the low task-specific prior knowledge instruction condition. Students in both the high and low task-specific prior knowledge instruction conditions studied their respective instructional materials for 20 min. All students were required to study, understand, and memorize the assigned content. In the present study, the task-specific prior knowledge manipulation should be understood as an experimentally induced, task-specific prior knowledge condition. It was designed to create different levels of immediate readiness for the subsequent polygon-area worked-example tasks. Similar experimental approaches have been used in previous collaborative learning research.

*Group assignment*: of the students who received high task-specific prior knowledge instruction, 67 participated in the subsequent experiment: 32 were assigned to the individual learning condition and 35 to the collaborative learning condition (comprising 11 triads and 1 dyad). Among those who received low task-specific prior knowledge instruction, 67 students were included, with 35 assigned to the individual condition and 32 to the collaborative condition (10 triads and 1 dyad).

Collaborative groups were primarily organized as triads. Triads were selected because they provide opportunities for peer explanation and shared processing while keeping communication and coordination demands manageable for fifth-grade students. This decision was also consistent with the cognitive-load perspective on collaboration, which suggests that the benefits of shared processing should be balanced against the transaction costs created by communication and coordination. Dong and Zhang (2018) found that, in worked-example learning, triads outperformed dyads and four-person groups on near-transfer performance for complex rule examples, which further supported the use of triads in the present study. Two dyads were formed because one student in each originally assigned triad was absent or did not complete the experimental procedure. Because the number of dyads was small and they were distributed across the prior knowledge conditions, they were retained in the analyses.

**Figure 1:**
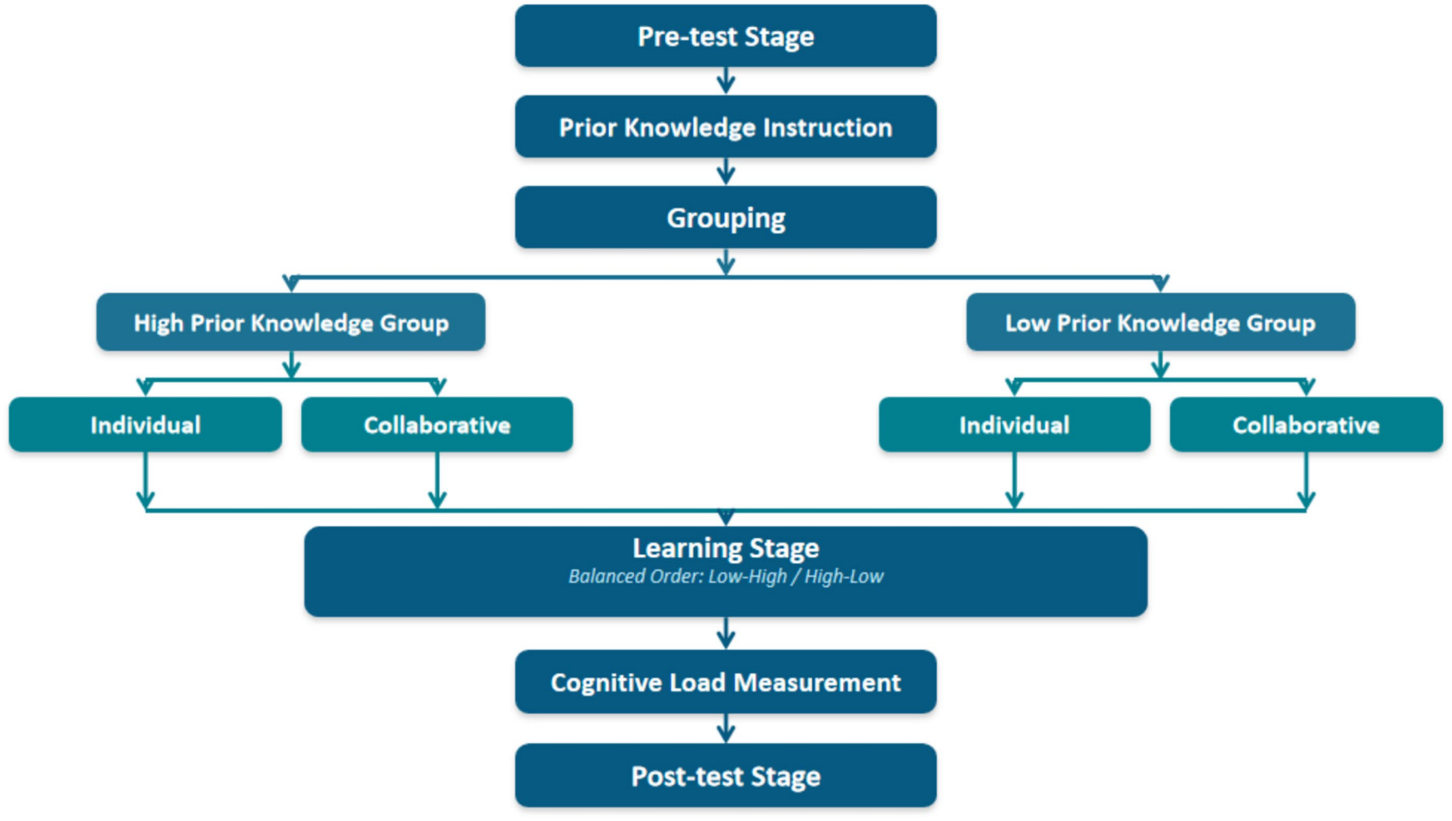
An overview of the procedure.

*Learning and testing*: task complexity was manipulated as a within-subjects factor, with all participants completing both low and high complexity worked-example tasks. To control for potential practice, fatigue, and order effects, the presentation order of tasks was counterbalanced: half of the participants completed the low complexity task first, followed by the high complexity task, while the other half completed the tasks in the reverse order. Participant distribution by task-specific prior knowledge level and learning condition was balanced across order conditions. Each of the four experimental groups completed the learning and testing procedures in separate classrooms under the experimenter’s supervision. In the low complexity condition, participants assigned to individual learning studied the materials independently for 15 min. Those in the collaborative learning condition worked together and jointly completed the formula derivation process for 15 min. During collaboration, all students were encouraged to participate actively, and off-task conversation was discouraged. Following the learning phase, all participants completed the near-transfer test, far-transfer test, and cognitive load measure within 20 min. In the high complexity condition, participants in the individual learning condition studied the materials independently for 20 min, while those in the collaborative condition studied and discussed the materials together for 20 min. As in the low complexity condition, students were encouraged to participate actively and avoid off-task conversation. After the learning phase, all participants completed the near-transfer test, far-transfer test, and cognitive load measure within 25 min. The two task complexity conditions were identical in instructions, procedures, and test requirements, differing only in the duration of learning and testing phases. The overall procedure is illustrated in Figure 1.

### 2.5 Scoring

Transfer tests were scored separately for the low and high complexity conditions, and all transfer scores were converted to percentages to facilitate comparisons across task conditions. The scoring rubrics were designed to ensure alignment between the test content and the target abilities assessed in this study. Specifically, students’ responses were not scored solely on the basis of final numerical answers; instead, points were awarded for correct formula application, key solution steps, and final calculation accuracy. This stepwise scoring approach was intended to capture students’ understanding of polygon-area formulas, their ability to apply appropriate solution procedures, and their capacity to transfer these procedures to new problem contexts.

*Low complexity condition*: for the low complexity condition, the near-transfer and far-transfer tests each comprised three items focused on calculating the areas of triangles, parallelograms, and trapezoids. Near-transfer items were structurally isomorphic to the worked examples. Each item was scored out of 3 points: 2 points for correctly applying the polygon area formula and 1 point for obtaining the correct answer, for a total of 9 points across three items. Far-transfer items were structurally non-isomorphic to the worked examples and also worth 3 points each: 2 points for correct application of the derived area formula and 1 point for the correct answer, totaling 9 points for three items.

*High complexity condition*: under the high complexity condition, the near-transfer and far-transfer tests each consisted of three items corresponding to triangle, parallelogram, and trapezoid area-calculation tasks. The near-transfer items were structurally isomorphic to the worked examples. Because the high-complexity tasks involved multiple solution steps, responses were scored stepwise. Each item was worth 6 points. At each key step, 2 points were awarded for correctly applying the polygon area formula and 1 point for obtaining the correct result, yielding a maximum score of 18 across the three items. The far-transfer items were structurally non-isomorphic to the worked examples and were scored in the same stepwise manner. Each item was worth 9 points. Across the solution steps, 2 points were awarded for correctly applying the polygon area formula and 1 point for obtaining the correct result, yielding a maximum score of 27 across the three items. To ensure scoring consistency, all responses were scored using a common rubric.

### 2.6 Data analysis

The main data collected in this study included near-transfer scores, far-transfer scores, and subjective cognitive load ratings. Transfer test scores were calculated separately for the low and high complexity task conditions and were converted into percentages. Subjective cognitive load was assessed using two 9-point self-report items measuring invested mental effort and perceived difficulty; these ratings were treated as indicators of learners’ subjective cognitive load.

Data were analyzed using repeated-measures analyses of variance (ANOVAs). Task-specific prior knowledge and learning condition were treated as between-subjects factors, and task complexity was treated as a within-subjects factor. Separate repeated-measures ANOVAs were conducted for near-transfer performance, far-transfer performance, and subjective cognitive load. When significant interactions were found, follow-up simple-effects analyses were conducted to examine differences between individual and collaborative learning within task-specific prior knowledge and task-complexity conditions. Partial eta squared (*η*^2^_p_) was reported as the measure of effect size. The significance level was set at *p* = 0.05.

## 3 Results

### 3.1 Near-transfer performance

Descriptive statistics for near-transfer performance in the four groups are shown in Table 1.

**Table 1:** Near-transfer scores (M ± SD) of individual and collaborative learning.

Task-specific prior knowledge	Low-complexity task condition		High-complexity task condition	
	Individual	Collaborative	Individual	Collaborative
High task-specific prior knowledge	60.07 ± 9.72	57.15 ± 12.96	45.31 ± 18.02	55.40 ± 13.64
Low task-specific prior knowledge	33.33 ± 21.56	41.32 ± 20.20	26.51 ± 16.73	31.08 ± 14.86

**Figure 2:**
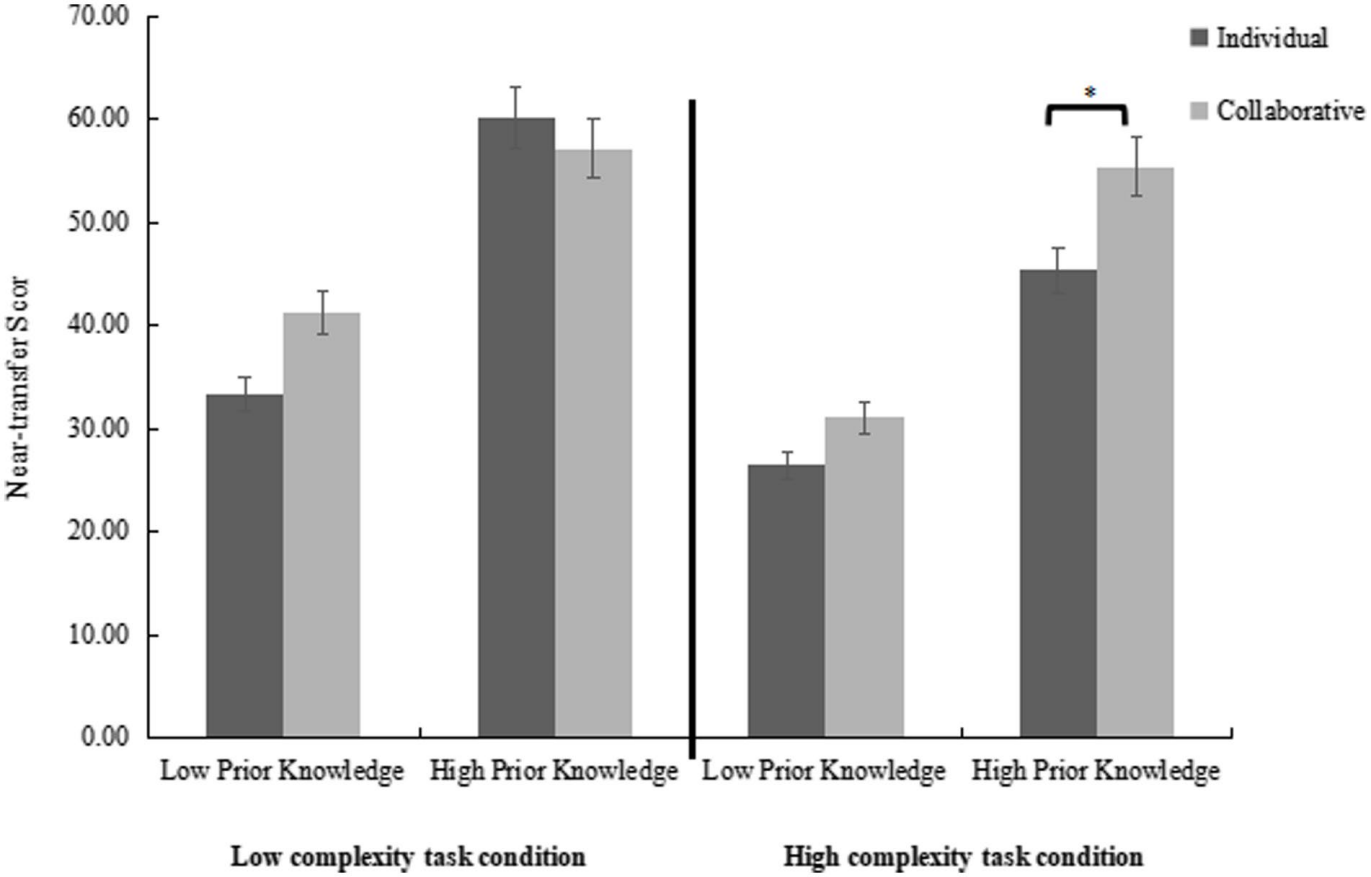
Near-transfer performance as a function of task-specific prior knowledge and task complexity under individual and collaborative learning conditions. Error bars represent standard errors. *p < 0.05.

Repeated-measures ANOVA revealed that, for near-transfer, the main effect of task-specific prior knowledge was significant, *F*(1, 130) = 87.30, *p* < 0.001, *η*^2^_p_ = 0.402. Significant main effects were also found for learning condition, *F*(1, 130) = 4.62, *p* < 0.05, *η*^2^_p_ = 0.034, and task complexity, *F*(1, 130) = 25.35, *p* < 0.001, *η*^2^_p_ = 0.163. Neither the interaction between task-specific prior knowledge and learning condition, *F*(1, 130) = 0.35, *p* > 0.05, *η*^2^_p_ = 0.003, nor the interaction between task complexity and task-specific prior knowledge, *F*(1, 130) = 0.01, *p* > 0.05, *η*^2^_p_ = 0.001, nor the interaction between task complexity and learning condition, *F*(1, 130) = 2.07, *p* > 0.05, *η*^2^_p_ = 0.016, was significant. By contrast, the three-way interaction among task-specific prior knowledge, learning condition, and task complexity was significant, *F*(1, 130) = 6.07, *p* < 0.05, *η*^2^_p_ = 0.045, indicating that the effect of learning condition on near-transfer performance depended jointly on task-specific prior knowledge and task complexity. Simple-effects analyses further showed that, among learners with high task-specific prior knowledge, collaborative learning did not differ significantly from individual learning under the low complexity condition, *F*(1, 65) = 1.08, *p* > 0.05, *η*^2^_p_ = 0.016. Under the high complexity condition, however, collaborative learning yielded significantly higher near-transfer performance than individual learning, *F*(1, 65) = 6.74, *p* < 0.05, *η*^2^_p_ = 0.094. Among learners with low task-specific prior knowledge, no significant differences between collaborative and individual learning were found under either task-complexity condition. The pattern of near-transfer results is shown in Figure 2.

### 3.2 Far-transfer performance

Descriptive statistics for far-transfer performance in the four groups are presented in Table 2.

**Table 2:** Far-transfer scores (M ± SD) of individual and collaborative learning.

Task-specific prior knowledge	Low-complexity task condition		High-complexity task condition	
	Individual	Collaborative	Individual	Collaborative
High task-specific prior knowledge	44.80 ± 19.65	47.30 ± 20.76	32.41 ± 20.95	46.24 ± 16.62
Low task-specific prior knowledge	19.05 ± 18.45	19.10 ± 16.75	15.23 ± 14.92	16.20 ± 14.72

**Figure 3:**
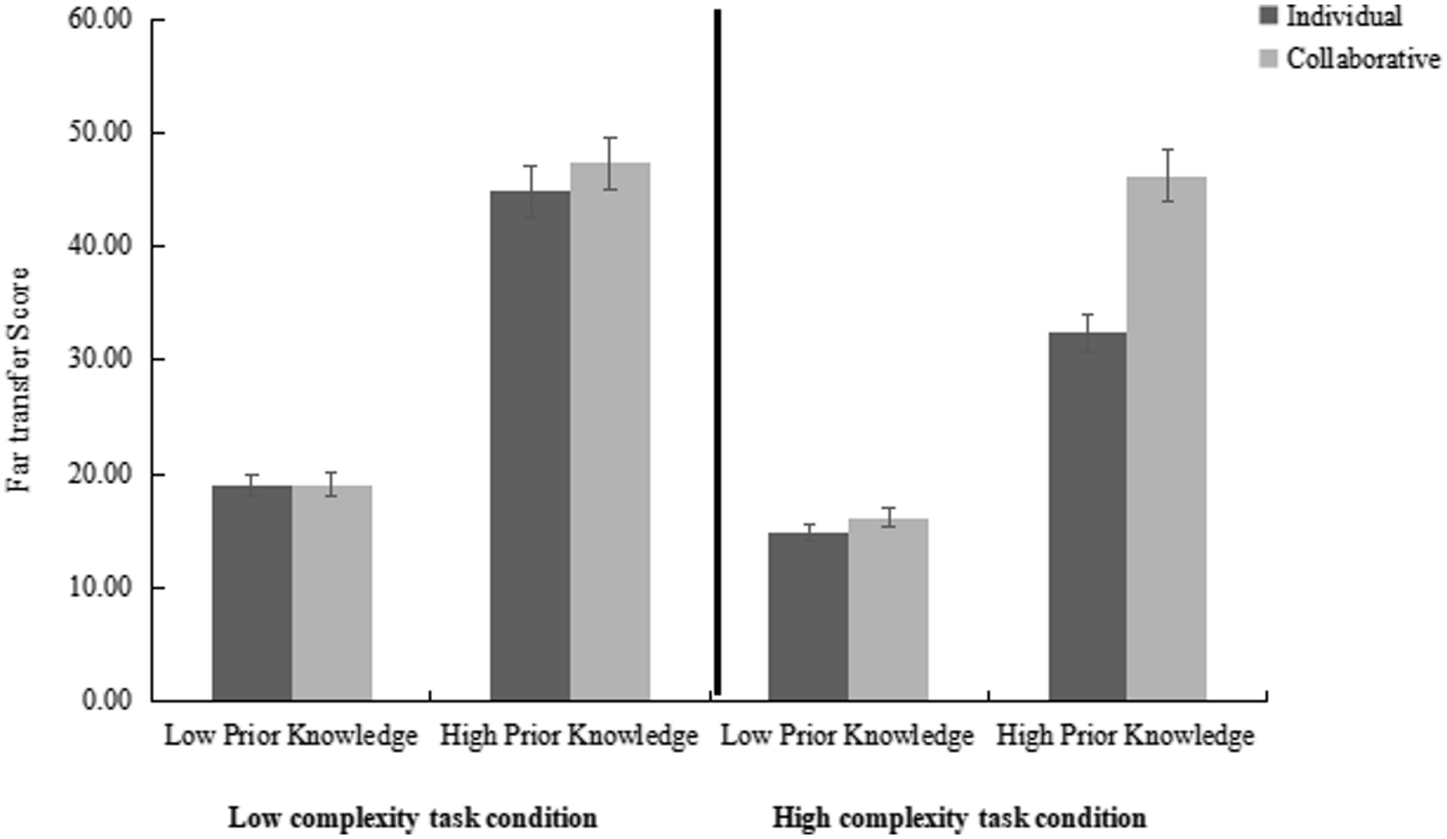
Far-transfer performance as a function of task-specific prior knowledge and task complexity under individual and collaborative learning conditions.

Repeated-measures ANOVA revealed that, for far-transfer, the main effect of task-specific prior knowledge was significant, *F*(1, 130) = 98.42, *p* < 0.001, *η*^2^_p_ = 0.431. The main effect of learning condition was not significant, *F*(1, 130) = 2.99, *p* > 0.05, *η*^2^_p_ = 0.022, whereas the main effect of task complexity was significant, *F*(1, 130) = 7.51, *p* < 0.01, *η*^2^_p_ = 0.055. Neither the interaction between task complexity and task-specific prior knowledge, *F*(1, 130) = 0.74, *p* > 0.05, *η*^2^_p_ = 0.006, nor the interaction between task-specific prior knowledge and learning condition, *F*(1, 130) = 2.15, *p* > 0.05, *η*^2^_p_ = 0.016, was significant. The interaction between task complexity and learning condition was not significant, *F*(1, 130) = 2.83, *p* > 0.05, *η*^2^_p_ = 0.021. The three-way interaction among task complexity, task-specific prior knowledge, and learning condition was not significant, *F*(1, 130) = 1.83, *p* > 0.05, *η*^2^_p_ = 0.014. The pattern of far-transfer results is shown in Figure 3.

### 3.3 Cognitive load

Descriptive statistics for cognitive load in the four groups are presented in Table 3.

**Table 3:** Cognitive load scores (M ± SD) of individual and collaborative learning.

Task-specific prior knowledge	Low-complexity task condition		High-complexity task condition	
	Individual	Collaborative	Individual	Collaborative
High task-specific prior knowledge	5.59 ± 1.25	4.58 ± 1.48	5.33 ± 1.37	5.11 ± 1.71
Low task-specific prior knowledge	5.29 ± 1.47	4.38 ± 1.33	5.80 ± 1.99	5.09 ± 1.92

**Figure 4:**
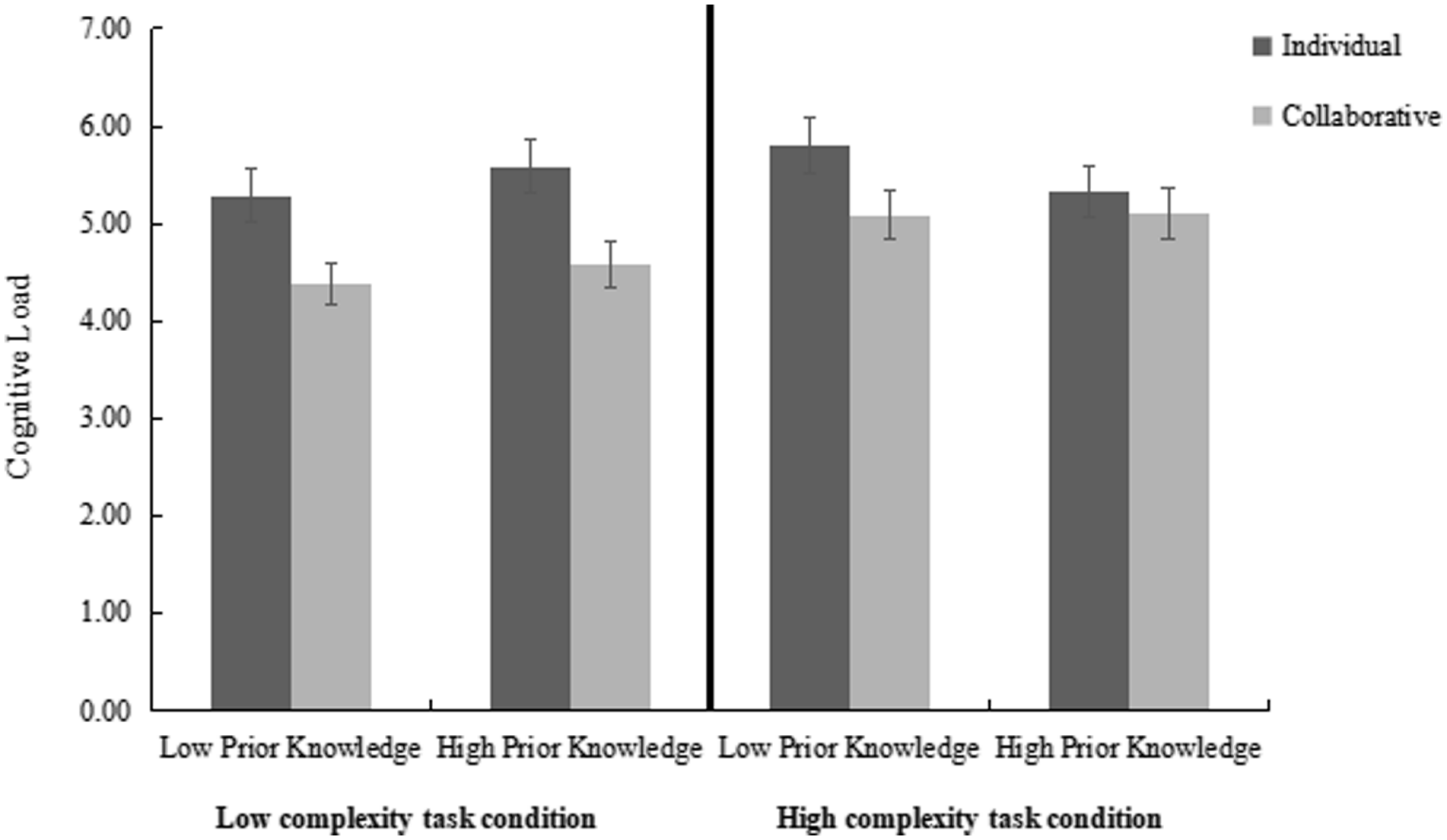
Cognitive load as a function of task-specific prior knowledge and task complexity under individual and collaborative learning conditions.

Repeated-measures ANOVA revealed that, for cognitive load, the main effect of task-specific prior knowledge was not significant, *F*(1, 130) = 0.002, *p* > 0.05, *η*^2^_p_ = 0.001. Significant main effects were found for learning condition, *F*(1, 130) = 8.74, *p* < 0.01, *η*^2^_p_ = 0.063, and task complexity, *F*(1, 130) = 8.03, *p* < 0.01, *η*^2^_p_ = 0.058. The interaction between task-specific prior knowledge and learning condition was not significant, *F*(1, 130) = 0.171, *p* > 0.05, *η*^2^_p_ = 0.001. The interaction between task complexity and task-specific prior knowledge was not significant, *F*(1, 130) = 3.05, *p* > 0.05, *η*^2^_p_ = 0.023. The interaction between task complexity and learning condition was not significant, *F*(1, 130) = 3.43, *p* > 0.05, *η*^2^_p_ = 0.026. The three-way interaction among task complexity, task-specific prior knowledge, and learning condition was not significant, *F*(1, 130) = 1.20, *p* > 0.05, *η*^2^_p_ = 0.009. The pattern of cognitive load results is shown in Figure 4.

## 4 Discussion

This study investigated how task-specific prior knowledge, learning condition, and task complexity interact in the context of learning from worked examples. The discussion below integrates findings on near-transfer, far-transfer, and cognitive load.

### 4.1 Near-transfer

The three-way interaction observed for near-transfer highlights an important boundary condition for the effectiveness of collaborative learning. Among learners with high task-specific prior knowledge, the benefits of collaboration were especially apparent in the high complexity condition. From a cognitive load theory perspective, high complexity tasks impose substantial intrinsic cognitive load, but learners with sufficient task-specific prior knowledge possess well-developed cognitive schemas to support task processing. In these situations, collaborative learning can help distribute cognitive load among group members through dialogue, explanation, and division of labor, creating a collective working memory that facilitates more efficient problem solving (Kirschner et al., 2009a). Additionally, from a social constructivist perspective, collaboration becomes a form of shared cognitive activity. When high task-specific prior knowledge learners explain solutions or discuss strategies with peers, they refine and reorganize their own thinking, which can deepen understanding and enhance near-transfer performance (Chi et al., 2008).

In contrast, learners with low task-specific prior knowledge showed only a slight advantage for collaborative learning over individual learning in the low complexity condition. This could be because low complexity tasks place modest demands on foundational knowledge, enabling collaboration to provide immediate support and opportunities for imitation. However, as task complexity increased, these learners may have experienced cognitive overload due to insufficient knowledge. In such cases, they may have struggled both to understand the task and to engage effectively in peer interaction, thus limiting the benefits of collaboration. Preparatory instruction created different levels of task-specific readiness. Learners with higher task-specific prior knowledge were better able to use collaboration to process high complexity worked examples, whereas learners with lower task-specific prior knowledge may have lacked the necessary conceptual and procedural foundation to benefit fully from peer interaction.

### 4.2 Far-transfer

In the far-transfer test, the main effects of task-specific prior knowledge and task complexity were both significant, indicating that learners’ level of task-specific prior knowledge and the complexity of the task itself were important factors influencing far-transfer performance. However, the main effect of learning condition and all interaction effects did not reach statistical significance. Descriptively, under the high complexity task condition, the collaborative learning group achieved higher far-transfer scores than the individual learning group, and this difference was more pronounced among learners with high task-specific prior knowledge. This pattern suggests that collaborative learning may have a potential advantage in complex far-transfer tasks. Far transfer requires learners to extract the underlying principles of a task and apply them to structurally different new contexts (Retnowati et al., 2018), and high complexity far-transfer tasks impose greater demands on cognitive processing. Collaborative learning may support learners’ identification of deep structures through peer explanation and multi-perspective discussion, which is consistent with the theoretical expectation of the collective working memory effect (Kirschner et al., 2011). However, because the interaction between task complexity and learning condition did not reach statistical significance, this possibility requires further verification in future research.

### 4.3 Cognitive load

The cognitive load results provided supplementary information for understanding the learning process. Specifically, the main effect of learning condition was significant, indicating that, overall, students in the collaborative learning condition reported lower subjective cognitive load than those in the individual learning condition. The main effect of task complexity was also significant, showing that high complexity tasks elicited higher subjective cognitive load than low complexity tasks overall. This finding is consistent with the basic assumptions of cognitive load theory: as the number of information elements that need to be processed and integrated simultaneously increases, learners are required to invest more cognitive resources. Overall, collaborative learning was associated with lower subjective cognitive load, whereas high complexity tasks were associated with higher subjective cognitive load. Theoretically, collaborative learning may reduce individual learners’ subjective load by distributing part of the cognitive processing demands through discussion, division of labor, and mutual explanation. In contrast, increased task complexity may raise the processing demands involved in integrating, reasoning about, and transferring problem information.

Taken together, these findings suggest that collaborative learning is not universally effective, but depends on the degree of fit between learners’ cognitive characteristics and task demands. The three-way interaction observed for near-transfer highlights this contextual boundary. When task complexity is high, and learners possess sufficient task-specific prior knowledge to support shared processing, collaboration is more likely to facilitate rule extraction and procedural reproduction through structured division of labor, mutual monitoring, and shared cognitive processing (Kirschner et al., 2009a). The far-transfer results were descriptively consistent with some theoretical expectations; however, because the relevant interaction effects did not reach statistical significance, they cannot be interpreted as confirmatory evidence for the proposed mechanism. The cognitive load results provide indirect support for the view that collaborative learning may reduce subjective cognitive load overall.

The findings of this study can be integrated into a relatively coherent explanatory framework. Learning condition and task complexity independently influenced students’ subjective cognitive load during learning, whereas task-specific prior knowledge further shaped whether learners could obtain observable learning benefits from collaboration. For learners with high task-specific prior knowledge, collaborative learning significantly facilitated near-transfer performance under high task complexity. This finding may suggest that, when learners possess the necessary foundational schemas, collaboration is more likely to support effective problem analysis, rule extraction, and procedural application. By contrast, for learners with low task-specific prior knowledge, although collaborative learning may reduce subjective cognitive load overall, this reduction in cognitive load did not translate into a stable transfer advantage. This suggests that lowering subjective cognitive load does not necessarily lead to better transfer performance. Whether learners possess sufficient knowledge foundations may be a critical prerequisite for their ability to allocate cognitive resources to deeper processing and knowledge transfer. Future research should further examine whether collaborative learning can produce stable advantages in complex far-transfer tasks and investigate, through collaborative process data, multidimensional measures of cognitive load, or mediation models, whether cognitive load plays an explanatory or mediating role in the effects of collaborative learning.

## 5 Limitations and future directions

Several limitations of this study should be acknowledged. First,this study manipulated experimentally induced task-specific prior knowledge via preparatory instruction, instead of grouping students based on their naturally acquired prior knowledge. Therefore, the high and low task-specific prior knowledge levels established in this study do not fully represent stable expertise and automated schemas stored in long-term memory in the strict sense of cognitive load theory. They only reflect varying degrees of learning readiness formed by experimental intervention when learners engage in subsequent worked-example learning tasks. Furthermore, although the preparatory phase was designed to induce differences in task-specific prior knowledge, future studies should further equate the amount and type of exposure to worked examples, procedural guidance, and practice opportunities across conditions to isolate the role of prior knowledge more precisely. Future research could use a pretest-based stratification approach to more directly examine the moderating role of naturally occurring prior knowledge, while retaining experimental manipulations of learning condition and task complexity. Second, task complexity was manipulated as a within-subjects variable. Although this design improved statistical power, repeated exposure to tasks of varying complexity may have introduced practice, fatigue, or order effects. Future studies could implement more rigorous counterbalancing or treat task complexity as a between-subjects variable to strengthen internal validity. Third, several transfer tests showed relatively low internal consistency. This issue may be partly attributable to the small number of test items and to the stepwise scoring rubric used in this study, in which scores were assigned separately for formula application, solution steps, and final answers. Insufficient reliability may reduce measurement precision and may also attenuate or obscure some experimental effects. Therefore, the transfer-related findings should be interpreted with caution. Future studies should increase the number of transfer test items and conduct further psychometric analyses to improve measurement reliability. Fourth, all participants were fifth-grade students, so the generalizability of these findings to other age groups or academic domains remains unclear. Future research could expand the sample’s age range to examine possible developmental variation. Finally, cognitive load was measured using subjective self-report. Although widely used, these measures are susceptible to individual biases. Future studies could combine physiological indicators with process-based measures to obtain more objective, multidimensional data on cognitive load, enabling a more precise analysis of cognitive resource allocation across experimental conditions.

## 6 Conclusion

The findings of this study indicate that the effectiveness of collaborative learning in elementary school mathematics using worked examples depends on both learners’task-specific prior knowledge and task complexity. For students with well-developed knowledge bases, collaboration is more likely to help them tackle complex tasks and facilitate knowledge transfer. In contrast, for students lacking sufficient knowledge preparation, the benefits of collaboration may not consistently appear.

Educational implications. In practice, collaborative learning should be used thoughtfully. Merely grouping students together does not guarantee deep learning. Instructional design should consider both task complexity and learners’ prior knowledge to create conditions for effective collaboration. Sufficient knowledge preparation and support before collaboration are especially important. Collaborative learning is most likely to be effective when learners have the necessary foundation and when task demands make collaboration valuable. Future instructional design and research should attend closely to this person-by-task dependency to maximize the effectiveness of collaborative learning.

## Data Availability

The original contributions presented in the study are included in the article/supplementary material, further inquiries can be directed to the corresponding author.
